# Metformin and insulin treatment of gestational diabetes: effects on inflammatory markers and IGF-binding protein-1 – secondary analysis of a randomized controlled trial

**DOI:** 10.1186/s12884-020-03077-6

**Published:** 2020-07-11

**Authors:** Mikael S. Huhtala, Kristiina Tertti, Juuso Juhila, Timo Sorsa, Tapani Rönnemaa

**Affiliations:** 1 Department of Obstetrics and Gynecology, University of Turku, 20014 Turku, Finland; 2 Department of Obstetrics and Gynecology, Turku University Hospital, Kiinamyllynkatu 4-8, FI-20521 Turku, Finland; 3 Medix Biochemica, Klovinpellontie 3, 02180 Espoo, Finland; 4 Department of Oral and Maxillofacial Diseases, Head and Neck Center, University of Helsinki and Helsinki University Hospital, P.O. Box 63, 00014 Helsinki, Finland; 5 Department of Dental Medicine, Karolinska Institute, Box 4064, 14104 Huddinge, Sweden; 6 Department of Medicine, University of Turku, 20014 Turku, Finland; 7 Department of Medicine, Turku University Hospital, Kiinamyllynkatu 4-8, 20521 Turku, Finland

**Keywords:** Gestational diabetes, Metformin, Low-grade inflammation, Insulin-like growth factor-binding protein 1, IGFBP-1

## Abstract

**Background:**

Gestational diabetes mellitus (GDM) is characterized by disturbed glucose metabolism and activation of low-grade inflammation. We studied whether metformin treatment has favorable or unfavorable effects on inflammatory markers and insulin-like growth factor-binding protein 1 (IGFBP-1) in GDM patients compared with insulin, and whether these markers associate with major maternal or fetal clinical outcomes.

**Methods:**

This is a secondary analysis of a previous randomized controlled trial comparing metformin (*n* = 110) and insulin (*n* = 107) treatment of GDM. Fasting serum samples were collected at the time of diagnosis (baseline, mean 30 gestational weeks [gw]) and at 36 gw. Inflammatory markers serum high-sensitivity CRP (hsCRP), interleukin-6 (IL-6), matrix metalloproteinase-8 (MMP-8) and glycoprotein acetylation (GlycA) as well as three IGFBP-1 phosphoisoform concentrations were determined.

**Results:**

In the metformin and insulin groups combined, hsCRP decreased (*p* = 0.01), whereas IL-6 (*p* = 0.002), GlycA (*p* < 0.0001) and all IGFBP-1 phosphoisoforms (p < 0.0001) increased from baseline to 36 gw. GlycA (*p* = 0.02) and non-phosphorylated IGFBP-1 (*p* = 0.008) increased more in patients treated with metformin than those treated with insulin. Inflammatory markers did not clearly associate with pregnancy outcomes but non-phosphorylated IGFBP-1 was inversely associated with gestational weight gain.

**Conclusions:**

Metformin had beneficial effects on maternal serum IGFBP-1 concentrations compared to insulin, as increased IGFBP-1 related to lower total and late pregnancy maternal weight gain. GlycA increased more during metformin treatment compared to insulin. The significance of this observation needs to be more profoundly examined in further studies. There were no evident clinically relevant relations between inflammatory markers and pregnancy outcome measures.

**Trial registration:**

The trial comparing metformin and insulin treatment was registered in ClinicalTrials.gov (NCT01240785) November 3, 2010. Retrospectively registered.

## Background

Gestational diabetes mellitus (GDM) is a growing health concern. It is associated with obesity and low-grade inflammation and increases the risk for pregnancy complications, such as macrosomia, preeclampsia, neonatal hypoglycemia and hyperbilirubinemia and the need for neonatal intensive care [1, 2]. In the long term GDM causes metabolic perturbations – it increases the risk for obesity and metabolic syndrome in the offspring [3] and the risk for type 2 diabetes (T2DM) in the mother [4]. Metformin treatment of GDM reduces gestational weight gain (GWG), gestational hypertension, the incidence of neonatal hypoglycemia and the need for neonatal intensive care compared to insulin treatment [5]. Although the benefits of metformin treatment during the pregnancy have been well characterized, there are concerns regarding the long term effects specially on the offspring [6]. Furthermore we do not know whether metformin has beneficial effects on low-grade inflammation compared to insulin.

Elevated serum IL-6 and high-sensitivity C-reactive protein (hsCRP) are markers of inflammation and predict the onset of T2DM [7]. Dysregulation of inflammation may be involved also in the pathogenesis of GDM [8]: hsCRP [9, 10], IL-6 [11] and glycoprotein acetylation (GlycA) [12] are related to GDM, and hsCRP also predicts the persistence of glucose intolerance postpartum [13]. Matrix metalloproteinase 8 (MMP-8), a more recent inflammatory marker, is related to intra-amniotic infection [14] and cervical ripening [15], but MMP-8 activity seems also to be increased in patients with GDM [16].

Besides inflammatory markers, a low serum concentration of insulin-like growth factor-binding protein 1 (IGFBP-1) is associated with GDM and an unfavorable metabolic profile [17]. IGFBP-1, in particular, is thought to play a significant role during pregnancy by regulating plasma glucose levels and being related to fetal growth [18]. Phosphorylation of IGFBP-1 increases its affinity to insulin like growth factor 1 (IGF-1). In the normal state, the highly phosphorylated isoform (high-pIGFBP-1) prevails, but during pregnancy, a non-phosphorylated IGFBP-1 (non-pIGFBP-1) is also detected. In cord blood, both phosphoisoforms are decreased in GDM and inversely associated to birth weight [19].

Based on earlier studies, metformin may have anti-inflammatory properties, as demonstrated by suppression of IL-6 (in vitro) [20] and hsCRP [21]. While insulin inhibits IGFBP-1 production [22], metformin appears to increase IGFBP-1 expression [23]. However, the possible effects of metformin on inflammatory markers in GDM pregnancy have not been studied in sufficiently large patient cohorts to give an unambiguous answer, and its effects on IGFBP-1 in GDM pregnancy have not been studied previously.

The primary aim of this study was to compare the effects of metformin and insulin treatment on the inflammatory markers hsCRP, IL-6, MMP-8, GlycA and three IGFBP-1 phosphoisoforms. The secondary aim was to examine whether variation in these variables at baseline (mean 30 gestational weeks, gw) or at late pregnancy (36 gw) are associated with the maternal and the neonatal outcomes. We hypothesized that metformin has beneficial effects on the inflammatory markers and IGFBP-1 compared to insulin.

## Methods

### Study design

The present study is a secondary analysis of a previous randomized trial [24], in which women with a singleton pregnancy and newly diagnosed GDM were treated either with metformin (*n* = 110) or insulin (*n* = 107) in an open-label randomized design. The original randomized trial was powered to prove non-inferiority of treatment to the primary outcome, which was birth weight. Since this was a secondary analysis, no power-analysis was made to calculate the sample size. However, an additional post-hoc power analysis is included as a supplementary file (Additional file 1). The patients were recruited at the Turku University Hospital on their first visit for management of GDM and they were randomized by the physician using sealed envelopes. GDM diagnosis was made based on the Finnish national guidelines and oral glucose tolerance test (OGTT) thresholds as described before [24]. Metformin treatment was started at a daily dose of 500 mg daily and increased up to 2000 mg if needed (median 1500 mg). Additional insulin was given to 23 participants in the metformin group due to unsatisfactory glucose control with metformin only.​ For insulin treatment, NPH insulin and/or rapid-acting insulin lispro or insulin aspart were used. The trial was approved by the Ethics Committee of the Southwest Hospital District of Finland, the Finnish National Agency of Medicines, and the European Union Drug Regulatory Agency (EUDRA) and registered retrospectively in ClinicalTrials.gov (NCT01240785, http://clinicaltrials.gov/ct2/show/NCT01240785). All participants provided written informed consent. The detailed design and outcomes of the randomized trial have been reported elsewhere [24].

For the present analysis Clinical data and serum samples from the previous randomized trial were available from 109 and 107 patients of the metformin and insulin groups, respectively. Those patients in the metformin group who received additional insulin are included in the metformin group unless otherwise specified.

### Biochemical methods and clinical variables

Fasting blood samples were drawn at baseline after the GDM diagnosis had been confirmed (mean 30 [20–34] gw) and at 36 gw. Serum concentrations of hsCRP and IL-6 were measured using ELISA [human C-reactive protein (CRP) ELISA kit, R&D Systems, Minneapolis, USA; interleukin-6 (IL-6) ELISA kit, R&D Systems, Minneapolis, USA]. MMP-8, non-pIGFBP-1, low-pIGFBP-1, high-pIGFBP-1 were determined using ELISA and an ﻿immunoenzymometric assay, as described earlier [15, 25], and GlycA according to a high-throughput proton (^1^H) nuclear magnetic resonance spectroscopy protocol [26].

Glucose values of the 2 h 75 g OGTT were available at the time of GDM diagnosis. C-peptide, HbA1c, age and pre-pregnancy BMI were assessed as risk factors for GDM and insulin resistance, to examine the relationship with the risk factors, the inflammatory markers and IGFBP-1’s. HbA1c was determined using high pressure liquid chromatography and fasting serum C-peptide by an electrochemiluminescence immunoassay. Both analytes were measured at baseline and HbA1c also at 36 gw.

Associations between inflammatory markers, IGFBP-1 phosphoisoforms and the following clinical outcomes were studied, A) maternal outcomes: GWG, preeclampsia or gestational hypertension, gestation length, induction of labor, incidence of cesarean section, and B) fetal outcomes: birth weight, neonate admission to NICU and neonatal intravenous glucose given for any indication. Total GWG was defined as the last measured weight at the maternity clinic minus self-reported weight before pregnancy, and late GWG as the weight gain from the initiation of antihyperglycemic medication. Birth weight was expressed in grams and in SD units (deviation from the mean value of the Finnish general population adjusted for gestation duration [27]). Birth weight > 90th percentile was used as an additional indicator of large for gestational age and a birth weight below <10th percentile was used to calculate the incidence of children of small for gestational age.

### Statistical analyses

Categorical clinical data comparison between groups was done with the χ^2^-test and Fisher’s exact test. Comparisons of means or medians was done using the Mann-Whitney U or t-test, depending on how the data was distributed. Wilcoxon’s test or the t-test was used for testing metabolite changes from baseline to 36 gw. An ANCOVA analysis was used to adjust for any differences between the compared groups. The normality of distributions was examined using the Shapiro-Wilk test when *n* < 100 and Kolmogorov-Smirnov’s test with Lilliefors’s correction for larger samples sizes. For correlations, Spearman’s rank correlation was used. For linear and logistic regression analyses, continuous variables were first centered and scaled, except for birth weight which already was expressed in terms of SD-units. Regression analyses were run both unadjusted and adjusted for treatment (metformin or insulin) and/or pre-pregnancy BMI, which was a priori thought to be the most clinically important confounding factor. Group-specific regression coefficients are given if the pharmacological treatment interacted significantly (*p* < 0.05) with the association between the independent and outcome variable in the regression model. Confidence intervals (CI) for regression coefficients were acquired with the adjusted bootstrap percentile method.

Results are reported with 95% CI; p < 0.05 was considered statistically significant. Bonferroni adjustment was applied on the regression analyses. Statistical analyses were run on the R statistics software (version 3.3.2, http://cran.r-project.org). This study adheres to CONSORT guidelines (http://www.consort-statement.org) for reporting clinical trials.

## Results

The study population characteristics are given in Table 1. Metformin and insulin groups were similar in terms of OGTT values, HbA1c at both time points, C-peptide, age, pre-pregnancy BMI and GWG. There were no differences in birth weight or proportion of primipara. There were no differences between the metformin and insulin groups regarding pregnancy outcomes, except for higher labor induction rates in the insulin group compared to the metformin group (54.2% vs. 37.6%, *p* = 0.014).

**Table 1:** Clinical characteristics of the study population

Variable	Metformin	n	Insulin	n	***p***-value
**Patients characteristics**					
Age (years)	31.9 ± 5.01	109	32.0 ± 5.47	107	0.89
Smoking	9 (8.6)	105	17 (16.0)	106	0.099
Primipara	42 (38.5)	109	49 (45.8)	107	0.28
Pre-pregnancy BMI (kg/m^2^)	29.5 ± 5.91	109	28.9 ± 4.71	107	0.41
**Glucose metabolism**					
HbA1c% at OGTT	5.48 ± 0.34	109	5.51 ± 0.34	107	0.49†
HbA1c at OGTT (mmol/mol)	36.3 ± 3.69		36.7 ± 3.72		
HbA1c% at 36 gw	5.68 ± 0.33	101	5.69 ± 0.36	95	0.82
HbA1c at 36 gw (mmol/mol)	38.5 ± 3.63		38.6 ± 3.89		
OGTT fasting (mmol/L)	5.52 ± 0.55	109	5.57 ± 0.42	107	0.44
OGTT 1 h (mmol/L)	11.2 ± 1.49	109	11.2 ± 1.24	107	0.61†
OGTT 2 h (mmol/L)	8.33 ± 1.76	108	7.91 ± 1.75	106	0.076
C-peptide at baseline (nmol/L)	1.05 ± 0.33	103	1.05 ± 0.29	101	0.90†
**Pregnancy outcomes**					
Gestational hypertension	2 (1.8)	109	4 (3.7)	107	0.44‡
Preeclampsia	5 (4.6)	109	10 (9.3)	107	0.19‡
Assisted vaginal delivery	9 (8.3)	109	8 (7.5)	107	0.83
Cesarean section	15 (13.8)	109	18 (16.8)	107	0.53
Induction of labor	41 (37.6)	109	58 (54.2)	107	0.014
Gestational weight gain (kg)	7.97 ± 5.24	108	7.82 ± 5.27	107	0.83
Weight gain in late gestation (kg)	1.79 ± 2.62	109	2.15 ± 2.97	107	0.35
Gw at delivery	39.2 ± 1.40	109	39.4 ± 1.58	107	0.43
**Neonatal outcomes**					
Birth weight (g)	3610 ± 490	109	3590 ± 450	107	0.78
Birth weight (SD)	0.17 ± 1.05	105	0.15 ± 0.96	107	0.91
Birth weight (centiles)	54.8 ± 28.9	105	54.3 ± 28.9	107	
Macrosomia	5 (4.6)	109	1 (0.9)	107	0.21‡
Birth weight < 10th percentile	12 (11.4)	105	9 (8.4)	107	0.46
Birth weight > 90th percentile	15 (14.3)	105	17 (15.9)	107	0.74
Admission to NICU	33 (30.1)	108	39 (36.4)	107	0.36
Newborn I.V. glucose	25 (23.1)	108	25 (23.6)	106	0.94

Data is shown as mean ± SD or n (%). The *p*-value is given for the t-test or the Mann-Whitney U (indicated with †) and for categorical data for the χ^2^-test or Fisher’s exact test (indicated with ‡). The number of mothers with clinical variables varied slightly due to missing data for some variables. OGTT = oral glucose tolerance test, gw = gestational weeks, SD = standard deviation, NICU = neonatal intensive care unit, I.V. = intravenous. Birth weight in SD and centiles were adjusted for Finnish population growth charts. Macrosomia was defined as birth weight > 4500 g or > 2 SD

### Inflammatory markers and IGFBP-1’s at baseline and change from baseline to 36 gw

Comparing metformin and insulin groups at baseline, there were no differences except for marginally lower low-pIGFBP-1 in the metformin group (21.0 vs. 24.0, *p* = 0.04). Within the metformin group, the inflammatory marker and IGFBP-1 concentrations did not differ when compared to those who required additional insulin treatment. Baseline and 36 gw values of the inflammatory markers and IGFBP-1’s are provided in detail in Additional file 2.

Changes in inflammatory markers and IGFBP-1 phosphoisoforms and comparison of changes are shown in Table 2. In the metformin and insulin groups combined, the hsCRP concentration decreased from baseline to 36 gw, whereas the IL-6, GlycA and IGFBP-1 concentrations increased. GlycA (*p* = 0.02) and non-pIGFBP-1 (*p* = 0.008) increased more in patients treated with metformin than with insulin but otherwise there were no statistically significant differences in these changes between the groups.

**Table 2:** Change in concentrations of inflammatory markers and IGFBP-1 phosphoisoforms from baseline to 36 gestational weeks

Variable	Metformin and insulin combined		Metformin		Insulin		***p***-value for comparison of changes (metf vs ins)
n	*179*		*94*		*85*		
	*median/mean (95% CI)*	*p-value*	*median/mean (95% CI)*	*p-value*	*median/mean (95% CI)*	*p-value*	
**Inflammation**							
hsCRP (mg/L)	−0.47 [−1.3; − 0.014]	0.011	− 0.45 [− 1.7; 0.16]	0.028	− 0.47 [− 1.8; 0.093]	0.18	0.72
IL-6 (ng/L)	0.70 [0.20; 1.40]	0.002	0.85 [0.50; 1.8]	0.002	0.62 [−0.19; 1.4]	0.13‡	0.31
MMP-8 (μg/L)	0.0 [−2.0; 0.80]	0.50	−0.70 [− 2.0; 1.0]	0.76	0.70 [− 2.0; 2.6]	0.20	0.28
GlycA (mmol/L)	0.11 [0.089; 0.13]	< 0.0001	0.15 [0.11; 0.18]	< 0.0001‡	0.091 [0.064; 0.12]	< 0.0001‡	0.020
**IGFBP-1**							
Non-phosphorylated (μg/L)	17.0 [13.0; 20.5]	< 0.0001	21.0 [14.0; 26.0]	< 0.0001	13.4 [7.9; 18.9]	< 0.0001‡	0.008
Low-phosphorylated (μg/L)	6.0 [4.0; 7.9]	< 0.0001	6.0 [3.6; 7.5]	< 0.0001	4.0 [−2.0; 4.0]	0.021	0.081
High-phosphorylated (μg/L)	300 [190; 410]	< 0.0001‡	260 [110; 420]	0.001‡	340 [180; 500]	< 0.0001‡	0.48†

Median/mean change from baseline to 36 gestational weeks [95% confidence interval (CI)]. Positive values indicate increase and negative values decrease. *p*-values are given for the one-sample t-test (indicated with ‡) or Wilcoxon’s signed rank test (comparisons not indicated by ‡). For comparison of changes between metformin and insulin groups, Mann-Whitney’s U-test or the t-test (indicated with †) was used. hsCRP = high sensitivity CRP, IL-6 = interleukin 6, MMP-8 = matrix metalloproteinase 8, GlycA = glycoprotein acetylation, IGFBP-1 = insulin-like growth factor-binding protein 1. n-values for GlycA are 190, 99 and 91 for combined, metformin and insulin groups, respectively

### Correlations between inflammatory markers, age, pre-pregnancy BMI and measures of glucose metabolism

**Fig. 1:**
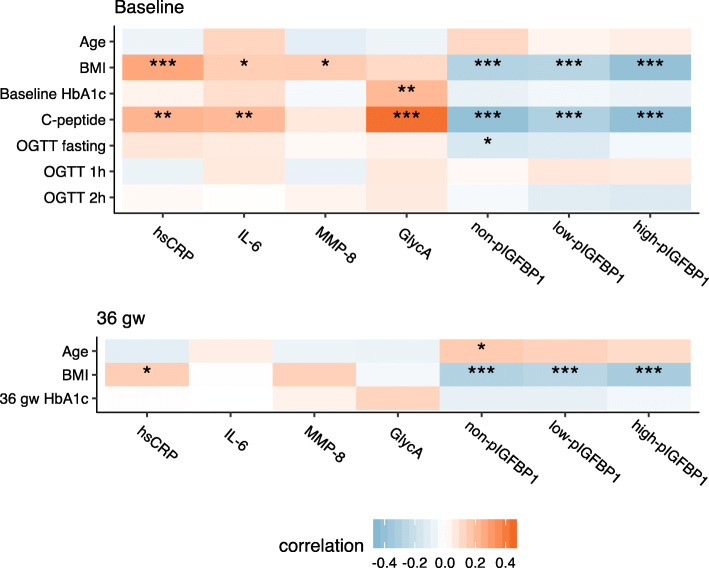
Heatmap representation of Spearman’s correlations between age, pre-pregnancy BMI and glucose metabolism with inflammatory markers and IGFBP-1 phosphoisoforms at baseline (*n* = 196–208) and at 36 gestational weeks (*n* = 181–198). BMI = body mass index, OGTT = oral glucose tolerance test, gw = gestational weeks, hsCRP = high sensitivity CRP, IL-6 = interleukin 6, MMP-8 = matrix metalloproteinase 8, GlycA = glycoprotein acetylation, non/low/high-pIGFBP-1 = non/low/high-phosphorylated insulin-like growth factor-binding protein 1. *p < 0.05, **p < 0.01, ***p < 0.001. This figure was created using *ggplot2* in R

Spearman’s correlations for inflammatory markers, IGFBP-1’s, age and variables related to pre-pregnancy BMI and glucose metabolism among the metformin and insulin treated patients are represented in Fig. 1. At baseline, hsCRP and IL-6 correlated positively and IGFBP-1 phosphoisoforms inversely with pre-pregnancy BMI and C-peptide. GlycA correlated at baseline with HbA1c and C-peptide but not with pre-pregnancy BMI. MMP-8 measured at baseline correlated only weakly with pre-pregnancy BMI.

### Regression analyses between inflammatory markers, IGFBP-1’s and clinical outcomes in metformin and insulin treated patients

#### Baseline

Non-pIGFBP-1 at baseline was associated with lesser total and late GWG (Table 3 and Additional file 3). After adjustment for pre-pregnancy BMI, both non-pIGFBP-1 (− 1.5 kg/SD, *p* < 0.0001) and low-pIGFBP-1 (− 0.99 kg/SD, *p* = 0.0037) were inversely associated with total GWG and non-pIGFBP-1 (− 0.47 kg/SD, *p* = 0.019) with late GWG (see Additional file 4 for adjusted regression results). Irrespective of these adjustments, MMP-8 was associated with late, but not total GWG. Only after adjustment for pre-pregnancy BMI, was hsCRP associated with total GWG (0.72 kg/SD, *p* = 0.05). HsCRP was positively associated with the gestation length and was not affected by adjustment for pre-pregnancy BMI (0.20 weeks/SD, *p* = 0.048). Non-pIGFBP-1 was associated with lower birth weight before (− 0.15 SD-unists/SD, *p* = 0.027) and after (− 0.14 SD-units/SD, *p* = 0.049) adjustment for pre-pregnancy BMI.

**Table 3:** Regression models with significant (*p* < 0.05) association of inflammatory markers and IGFBP-1 concentrations with maternal and neonatal outcomes

Independent variable	Outcome	β-estimate [95% CI] (***p***-value)	n total
**Baseline**			
non-pIGFBP-1	total GWG (kg/SD)	−1.2 [− 2; −0.64] (< 0.001)*	201
MMP-8	late GWG (kg/SD)	0.41 [0.022; 0.77] (0.035)	202
non-pIGFBP-1	late GWG (kg/SD)	0.45 [−0.87; − 0.13] (0.021)	202
hsCRP	length of gestation (weeks/SD)	0.2 [0.028; 0.36] (0.044)	202
high-pIGFBP-1	induction of labor (OR/SD)†	0.67 [0.48; 0.92] (0.0094)	202
non-pIGFBP-1	birth weight (SD/SD)	−0.15 [−0.32; − 0.052] (0.027)	198
**36 gestational weeks**			
non-pIGFBP-1	total GWG (kg/SD)	−1.1 [−1.8; −0.52] (0.0027)*	188
non-pIGFBP-1	late GWG (kg/SD)	−0.55 [− 0.96; − 0.21] (0.0069)	189
non-pIGFBP-1	cesarean section (OR/SD)‡	0.49 [0.24; 0.84] (0.043)	189
MMP-8	birth weight (SD/SD)	−0.17 [− 0.34; − 0.037] (0.022)	185

Both metformin and insulin treated patients were included. Induction of labor was performed in 92 and cesarean section in 26 women. Data is given as regression β-estimates or odds ratios (OR) in respect to one SD change of the predictor [95% confidence interval, CI] (*p*-value). The reference groups for binary outcomes were no induction of labor (†) and vaginal delivery (‡). SD = standard deviation, GWG = (maternal) gestational weight gain, pIGFBP-1 = phosphorylated insulin-like growth factor-binding protein 1, MMP-8 = matrix metalloproteinase 8, hsCRP = high sensitivity CRP. *p < 0.0045 (Bonferroni)

#### Gestational week 36

Similarly to baseline, non-pIGFBP-1 measured at 36 gw was associated with lesser total and late GWG, and after adjustment for pre-pregnancy BMI also low-pIGFBP-1 was associated with total GWG. In the metformin group, MMP-8 was related to higher late GWG (0.74 kg/SD, *p* = 0.35) and hsCRP with longer gestation (0.40 weeks/SD, *p* = 0.046), and these associations were unaffected by adjustment for pre-pregnancy BMI. A high non-pIGFBP-1 concentration was related to a lower incidence for cesarean section (OR: 0.49, *p* = 0.043), but this association was no longer significant after adjustment for pre-pregnancy BMI. A high MMP-8 was associated with lower birth weight (− 0.17 SD-units/SD, *p* = 0.022), and this association was not affected by pre-pregnancy BMI.

When the regression *p*-values at each time point were adjusted using Bonferroni method, the associations between non-pIGFBP-1 and GWG remained significant at both time points in models irrespective of adjustment for pre-pregnancy BMI. In addition the association between low-pIGFBP-1 at baseline and total GWG was significant in the regression adjusted for pre-pregnancy BMI. Regression results for metformin and insulin groups separately are shown in Additional file 5, for those models in which there was a significant interaction (*p* < 0.05) in the association between the independent and outcome variable. None of the *p*-values for metformin and insulin groups separately reached Bonferroni adjusted threshold of *p* < 0.0045.

## Discussion

Seven biomarkers at the time of GDM diagnosis and at 36 gestational weeks were analyzed and the effects of metformin and insulin treatment on the biomarker concentrations and their relation to clinical outcomes were compared. In addition to the traditional markers hsCRP and IL-6, also MMP-8 and GlycA were included in the analyses, since both of these markers are promising markers of cardiovascular risk outside pregnancy [28, 29].

In both treatment groups hsCRP decreased from baseline to 36 gw, as demonstrated previously in non-diabetic obese and normal-weight pregnant women [30]. To our knowledge, this is the largest sample comparing the effect of metformin and insulin on hsCRP in GDM. In another large trial comparing metformin and insulin treatment in GDM (the MiG trial), CRP remained unchanged from GDM diagnosis to 36 gw [31]. Notwithstanding the different quantification method, this difference may be explained by lower baseline hsCRP in the MiG study [31]. Conversely, hsCRP has been related to BMI [32], which was higher in MiG than in our cohort; this emphasizes the possible effects of ethnicity and the need for absolutely identical diagnostic criteria for GDM.

In line with previous reports in non-diabetic subjects, IL-6 increased during the last trimester of pregnancy [30]. IL-6 is secreted to a large extent by adipocytes and correspondingly higher serum concentrations are associated with higher BMI [30]. However, IL-6 has also anti-inflammatory effects [33] and considering the lack of associations with any adverse outcomes in our data, the complexity of IL-6 signaling in pregnancy remains incompletely understood. Still, we have demonstrated that compared with insulin metformin treatment of GDM does not appear to affect serum IL-6.

Previously it has been shown that, in the presence of premature rupture of membranes, maternal serum IL-6 predicts preterm delivery at 72 h before delivery [34]. In our data there was an inverse, albeit statistically non-significant association between IL-6 at 36 gw and gestation length.

Serum GlycA increased in both treatment groups but more in response to metformin treatment. This is in contrast to a previous study in non-diabetic individuals where metformin did not affect serum GlycA [35]. However, the serum concentrations of some glycoproteins, such as α-1-acid glycoprotein and α-1-antitrypsin, change in normal pregnancy [36], and this confuses the interpretation of GlycA. In general, pregnancy is associated with activation of the innate immune system and with an increase in the concentration of acute phase proteins in the serum. An overall increase of GlycA during pregnancy has been reported previously in a population cohort study [37] and this probably reflects changes in the immune system [38]. High GlycA predicts T2DM [39] and cardiovascular [29] risk in non-pregnant women. Similarly, in pregnancy it has been associated with insulin resistance, a poor lipid profile [40] and GDM in obese women [12]. In agreement with this, GlycA correlated with HbA1c and C-peptide at baseline but not with HbA1c at 36 gw. These results suggest that GlycA may not be a reliable marker of inflammation near term, possibly due to changes in glycoprotein composition [36].

Serum MMP-8 was rather constant during the last trimester of pregnancy, and to our knowledge this is the first longitudinal study characterizing MMP-8 in GDM. Outside GDM, MMP-8 is associated with chorioamnionitis [14] and preterm delivery [41]. Although we did not observe an association between maternal serum MMP-8 and gestation length, MMP-8 was associated with a slightly reduced birth weight. Serum MMP-8 may indicate subclinical inflammation of the placenta or the chorion, which would affect birth weight.

In normal pregnancy, serum IGFBP-1 increases during the first trimester and then decreases slightly before another peak just before delivery [42]. In our data, IGFBP-1 phosphoisoform concentrations increased from baseline to 36 gw in both treatment groups. Non-pIGFBP-1 concentrations increased significantly more in women treated with metformin, and there was a trend towards a higher concentration of low-pIGFBP-1. In line with this, metformin causes a marked increase in IGFBP-1 in non-pregnant women with the polycystic ovary syndrome [43]. Metformin increases insulin sensitivity and this might decrease insulin levels. There is a negative feedback loop from insulin to the production of IGFBP-1 [22], and this might explain the difference in serum IGFBP-1 levels between the treatment groups. Another possibility is that the increase in IGFBP-1’s in the metformin group is a consequence of dietary changes in response to gastrointestinal symptoms often occurring during metformin use. Previously metformin treatment has been related to lower GWG when compared to either insulin [44] or placebo [45]. And although in our data there were no differences in GWG between the treatment groups, non-pIGFBP-1 and low-pIGFBP-1 were inversely associated with GWG.

Neither at baseline nor at 36 gw was there any apparent association between inflammatory markers, IGFBP-1’s and clinical outcomes, with the exception of the inverse association between non-pIGFBP-1, low-pIGFBP-1 and GWG.

IGFBP-1 phosphoisoform concentrations were associated with healthier metabolic profiles, as expected, but high non-pIGFBP-1 and low-pIGFBP-1 were also related to lesser GWG. High pre-pregnancy BMI and high GWG are two major risk factors of excessive fetal growth. In spite of that, IGFBP-1’s in our data were not clearly associated with any birth weight variables. This is in contrast with previous results from a population cohort where low IGFBP-1 throughout pregnancy was related with a higher birth weight [46]. The discrepancy may at least in part be explained by the fact that our study population, having GDM and being therefore at risk for fetal macrosomia, were given intensive dietary and lifestyle counselling after the GDM diagnosis to prevent excessive weight gain.

Metformin has been found to reduce the risk of gestational hypertension in comparison to insulin [5] and the risk of preeclampsia when compared to placebo [45]. This effect however was unlikely mediated by reduction of insulin resistance in obese patients [47]. In line with these findings, neither IGFBP-1’s nor the inflammatory markers were associated with the risk of hypertensive disorders in our data.

Baseline high-pIGFBP-1 in all patients requiring metformin or insulin and low-pIGFBP-1 in metformin-treated patients was associated with a lower risk for induction of labor. This may reflect a better overall metabolic health of patients with high serum IGFBP-1 while having a lower overall risk for pregnancy complications (of which induction of labor was the most frequent). The induction rate of labor was marginally higher in patients treated with insulin. This might reflect the physicians’ perception that GDM treated with insulin is more severe than GDM without insulin treatment.

In our study, at baseline the inflammatory markers hsCRP, IL-6 and GlycA, and IGFBP-1 phosphoisoforms correlated stronger with fasting C-peptide and pre-pregnancy BMI than with fasting or postprandial glucose. Hence, inflammatory markers and IGFBP-1 phosphoisoforms seem to indicate obesity related insulin resistance.

We have demonstrated that metformin affects serum GlycA and non-pIGFBP-1 in GDM, and that the associations between these markers and clinical outcomes are similar irrespective of the antihyperglycemic treatment used. Based on this data it is unlikely that metformin, at least when started this late in pregnancy, has any significant impact on the systemic low-grade inflammation that is present in GDM [9–12] or reflects morbidity later in life [13]. Follow-up studies are needed to assess the long term safety of metformin treatment of GDM on children. Further on, it needs to be studied whether possible long term consequences are associated with the changes in serum inflammatory markers or IGFBPs.

### Strengths and limitations of the study

We have included two relatively novel inflammatory markers, MMP-8 and GlycA, and provide longitudinal data of their changes during the last trimester of pregnancy. The study design was a randomized controlled trial – a setting that improves the reliability of results. Even so, there are some limitations to our study.

Our sample size was designed to prove non-inferiority of metformin or insulin in birth weight in the previously published primary randomized trial (24). Thus, although the study population is fairly large, it was underpowered to reveal or exclude all studied associations between inflammation markers and IGFBP-1 s and outcome variables. There may also be confounding factors that slightly affect both maternal and neonatal outcomes, but the statistical power of multiple adjusted regression models to examine each outcome closely is limited. The serum samples in late pregnancy were taken at mean 36 gw of the patients. Since the women delivered at mean 39 gw, additional samples taken nearer delivery could have provided important additional information on the effect of metformin and insulin. Our population is representative of mostly Caucasian patients in excellent glycemic control, and these results may not necessarily be generalizable to populations of other ethnicities or with inferior glycemic control. Furthermore the indications for induction, cesarean section and NICU admissions vary between countries making comparisons of these outcomes between various studies difficult. The trial was registered at ClinicalTrials.gov retrospectively.

## Conclusions

Metformin had beneficial effects on maternal serum IGFBP-1 concentrations compared to insulin, possibly due to its favorable effect on insulin resistance. IGFBP-1, the non-phosphorylated isoform in particular, related to lower total and late pregnancy maternal weight gain. Otherwise there were no evident clinically relevant relations between inflammatory markers and pregnancy outcome measures. Compared to insulin metformin caused a similar decrease in serum hsCRP and a similar increase in IL-6 but a slightly greater rise in GlycA. The significance of GlycA, and of IL-6-CRP-signalling in GDM will need to be more profoundly examined in further studies.

## Supplementary information

**Additional file 1.** Post-hoc power analysis.

**Additional file 2: Table S1.** Comparison of inflammatory markers and IGFBP-1’s at baseline and at 36 gestational weeks.

**Additional file 3: Table S2.** Associations of inflammatory markers and IGFBP-1 concentrations with clinical outcomes.

**Additional file 4: Table S3.** Associations of inflammatory markers and IGFBP-1 concentrations at baseline and 36 gestational weeks with clinical outcomes adjusted for pre-pregnancy BMI in metformin and insulin treated patients combined.

**Additional file 5: Table S4.** Regression models with significant (*p* < 0.05) interaction between treatment group (metformin or insulin) and the association between outcome and the independent variable.

## Data Availability

The datasets used and/or analysed during the current study are available from the corresponding author on reasonable request.

## Abbreviations

BMI: Body mass index
CI: Confidence interval
CRP: C-reactive protein
hsCRP: High-sensitivity C-reactive protein
ELISA: Enzyme-linked immunosorbent assay
FDR: False discovery rate
GDM: Gestational diabetes mellitus
GlycA: Glycoprotein acetylation
Gw: Gestational weeks
GWG: Gestational weight gain
HbA1c: glycated hemoglobin
IGF-1: Insulin-like growth factor 1
IGFBP-1: (non-pIGFBP-1, low-pIGFBP-1, high-pIGFBP-1) non-phosphorylated / low-phosphorylated / high-phosphorylated insulin-like growth factor-binding protein 1
IL-6: Interleukin-6
LDL: Low-density lipoprotein
MMP-8: Matrix metalloproteinase-8
NICU: Neonatal intensive care unit
OGTT: Oral glucose tolerance test
SD: Standard deviation
T2DM: Type 2 diabetes mellitus
