# Supplementary material for: Metformin and insulin treatment of gestational diabetes: effects on inflammatory markers and IGF-binding protein-1 – secondary analysis of a randomized controlled trial
Source: BMC Pregnancy Childbirth. 2020 Jul 11;20:401. doi: 10.1186/s12884-020-03077-6 (PMC7353798; doi:10.1186/s12884-020-03077-6)
Supplement: Supplementary file 1 — Additional file 1. Post-hoc power analysis. [file 12884_2020_3077_MOESM1_ESM.docx]

Supplementary file

Post-hoc power analyses

In order to estimate the sample size needed for future studies, we performed a post-hoc power analysis of the main outcome measures: the differences of inflammatory marker and IGFBP-1 phosphoisoform changes (from 30 to 36 gestational weeks) between metformin and insulin groups, and their association with clinical outcomes. For the sake of simplicity, we calculated the regression sample size only for the primary outcome of the original randomized controlled trial, which was birth weight.

The power analysis was performed for Mann-Whitney U test or t-test depending of which test was used in our analyses. For Mann-Whitney U test we assumed similar data distribution as in our data.

The analyses were performed in R statistics software (version 3.6.1) using *samplesize* and *pwr* packages.

Sufficient total sample sizes to prove significant difference in changes (from baseline to 36 gestational weeks) between metformin and insulin group in Mann-Whitney U test with two-sided alpha of 0.05 and power of 0.80 would be: hsCRP n=15500, IL-6 n=1230, MMP-8 n=1490, GlycA n=272, non-pIGFBP-1 n=205, and low-pIGFBP-1 n=452, respectively. For high-pIGFBP-1 a sufficient sample size to prove 50% difference in mean change using t-test with similar alpha and power, would be n=776.

The sample size required to prove significant association between any given biomarker and birth weight in all patients combined in unadjusted regression analysis with R^2^ of 2%, alpha of 0.05 and power of 0.80 would be n=387.
