# Supplementary material for: Metformin and insulin treatment of gestational diabetes: effects on inflammatory markers and IGF-binding protein-1 – secondary analysis of a randomized controlled trial
Source: BMC Pregnancy Childbirth. 2020 Jul 11;20:401. doi: 10.1186/s12884-020-03077-6 (PMC7353798; doi:10.1186/s12884-020-03077-6)
Supplement: Supplementary file 2 — Additional file 2: Table S1. Comparison of inflammatory markers and IGFBP-1’s at baseline and at 36 gestational weeks. [file 12884_2020_3077_MOESM2_ESM.docx]

**Supplementary table 1 – Comparison of inflammatory markers and IGFBP-1’s at baseline and at 36 gestational weeks**

| **Variable** | | **Baseline** | | **p-value** |  | **36 Gestational Weeks** | | **p-value** |
| --- | --- | --- | --- | --- | --- | --- | --- | --- |
|  | | **Metformin** | **Insulin** |  |  | **Metformin** | **Insulin** |  |
| n: | | 107 | 95 |  |  | 95­ | 94 |  |
| **Inflammation** | |  |  |  |  |  |  |  |
|  | hsCRP (mg/L) | 10.1 [2.9–15.4] | 11.4 [4.2–19.4] | 0.37 |  | 8.0 [2.7–12.9] | 8.7 [4.6–15.7] | 0.33 |
|  | IL-6 (ng/L) | 2.1 [0.95–4.8] | 2.8 [1.3–4.8] | 0.46 |  | 2.9 [1.9–6.9] | 3.3 [1.8–5.8] | 0.58 |
|  | MMP-8 (µg/L) | 14.0 [9.9–23.0] | 15.0 [10.0–20.5] | 0.89 |  | 15.0 [8.8–22.5] | 16.0 [10.0–25.0] | 0.19 |
|  | GlycA (mmol/L) | 1.71 [1.64–1.82] | 1.76 [1.63–1.87] | 0.22 |  | 1.83 [1.75–1.98] | 1.80 [1.70–1.94] | 0.24 |
| **IGFBP-1** | |  |  |  |  |  |  |  |
|  | Non-phosphorylated (µg/L) | 73.0 [42.0–98.5] | 82.0 [49.0–120.0] | 0.12 |  | 95.0 [65.5–132.0] | 97.5 [59.3–131.8] | 0.93 |
|  | Low-phosphorylated (µg/L) | 21.0 [15.0–33.0] | 24.0 [16.5–39.0] | 0.041* |  | 27.0 [21.0–39.5] | 29.5 [19.0–45.0] | 0.59 |
|  | High-phosphorylated (µg/L) | 1210 [814–1830] | 1150 [808–1690] | 0.64 |  | 1380 [1050–2050] | 1560 [1180–1970] | 0.44 |

Values are given as median [interquartile range] serum concentration. p-value is given for the Mann-Whitney U test. n for GlycA at baseline were 108 and 100, and at 36 gestational weeks 100 and 98 for metformin and insulin groups, respectively. hsCRP = high sensitivity CRP, IL-6 = interleukin 6, MMP-8 = matrix metalloproteinase 8, GlycA = glycoprotein acetylation, IGFBP-1 = insulin-like growth factor-binding protein 1
