# Supplementary material for: Metformin and insulin treatment of gestational diabetes: effects on inflammatory markers and IGF-binding protein-1 – secondary analysis of a randomized controlled trial
Source: BMC Pregnancy Childbirth. 2020 Jul 11;20:401. doi: 10.1186/s12884-020-03077-6 (PMC7353798; doi:10.1186/s12884-020-03077-6)
Supplement: Supplementary file 3 — Additional file 3: Table S2. Associations of inflammatory markers and IGFBP-1 concentrations with clinical outcomes. [file 12884_2020_3077_MOESM3_ESM.docx]

**Supplementary table 2a – Associations of inflammatory markers and IGFBP-1 concentrations at baseline with maternal and neonatal outcomes**

|  | | **Maternal outcomes** | | | | | |  | **Neonatal outcomes** | | | | |
| --- | --- | --- | --- | --- | --- | --- | --- | --- | --- | --- | --- | --- | --- |
|  | | **Total gestational weight gain** | **Late gestational weight gain** | **Preeclampsia or gestational hypertension** | **Length of gestation** | **Induction of labor** | **Cesarean section** |  | **Birth weight** | **Birth weight <10^th^ percentile** | **Birth weight >90^th^ percentile** | **NICU admission** | **Newborn I.V. glucose** |
|  |  | *kg/SD* | *kg/SD* | *OR/SD* | *weeks/SD* | *OR/SD* | *OR/SD* |  | *SD/SD* | *OR/SD* | *OR/SD* | *OR/SD* | *OR/SD* |
|  | total n:  n with event: | *201* | *202* | *202*  *17* | *202* | *202*  *92* | *202*  *29* |  | *198* | *198*  *17* | *198*  *30* | *201*  *67* | *200*  *45* |
| **Inflammation at baseline** | |  |  |  |  |  |  |  |  |  |  |  |  |
|  | hsCRP | 0.25 [-0.39; 0.87] | 0.19 [-0.25; 0.6] | 1 [0.54; 1.6] | 0.2 [0.028; 0.36]* | 0.84 [0.63; 1.1] | 1.1 [0.72; 1.5] |  | -0.047 [-0.19; 0.086] | 1.2 [0.69; 2] | 0.86 [0.58; 1.2] | 0.95 [0.66; 1.3] | 1.1 [0.69; 1.5] |
|  | IL-6 | -0.011 [-0.66; 0.7] | -0.066 [-0.49; 0.39] | 1.1 [0.7; 1.7] | 0.013 [-0.17; 0.18] | 0.82 [0.61; 1.1] | 1.1 [0.73; 1.6] |  | 0.024 [-0.1; 0.16] | 0.88 [0.44; 1.4] | 1 [0.73; 1.5] | 1 [0.74; 1.4] | 1 [0.75; 1.4] |
|  | MMP-8 | 0.012 [-0.71; 0.69] | 0.41 [0.022; 0.77]* | 1.3 [0.63; 2.2] | 0.021 [-0.17; 0.21] | 1 [0.74; 1.3] | 1.4 [0.97; 2] |  | -0.016 [-0.14; 0.11] | 0.99 [0.6; 1.6] | 0.88 [0.54; 1.4] | 1.2 [0.84; 1.6] | 1.2 [0.8; 1.6] |
|  | GlycA | -0.14 [-0.87; 0.58] | -0.32 [-0.74; 0.064] | 1.5 [0.97; 2.4] | -0.18 [-0.35; 0.0064] | 1.2 [0.88; 1.5] | 1.2 [0.87; 1.7] |  | 0.039 [-0.1; 0.18] | 1.1 [0.7; 2] | 0.93 [0.65; 1.3] | 1.1 [0.79; 1.4] | 1.1 [0.82; 1.6] |
| **IGFBP-1 at baseline** | |  |  |  |  |  |  |  |  |  |  |  |  |
|  | Non-phosphorylated | -1.2 [-2; -0.64]**# | -0.45 [-0.87; -0.13]* | 0.89 [0.36; 1.9] | 0.068 [-0.21; 0.26] | 0.88 [0.53; 1.2] | 0.53 [0.24; 0.93] |  | -0.15 [-0.32; -0.052]* | 1.1 [0.66; 2.2] | 0.55 [0.28; 0.97] | 1.2 [0.77; 1.6] | 1.1 [0.62; 1.6] |
|  | Low-phosphorylated | -0.72 [-1.4; 0.036] | -0.35 [-0.66; -0.017] | 0.94 [0.38; 1.7] | -0.041 [-0.28; 0.16] | 0.84 [0.57; 1.1] | 0.79 [0.39; 1.3] |  | -0.071 [-0.19; 0.044] | 1 [0.59; 1.6] | 0.66 [0.37; 1] | 0.89 [0.62; 1.2] | 0.87 [0.53; 1.3] |
|  | High-phosphorylated | 0.19 [-0.56; 0.97] | -0.32 [-0.63; 0.036] | 0.76 [0.44; 1.2] | -0.056 [-0.25; 0.13] | 0.67 [0.48; 0.92]** | 0.71 [0.44; 1.1] |  | -0.027 [-0.15; 0.098] | 0.74 [0.42; 1.2] | 0.77 [0.49; 1.1] | 1 [0.75; 1.4] | 0.99 [0.69; 1.4] |

Both metformin and insulin treated patients were included. Measures are expressed as odds ratios (OR) or regression β-estimates with 95% confidence intervals. Birth weight was measured in population SD units. SD = standard deviation, NICU = neonatal intensive care unit, i.v. = intravenous, hsCRP = high sensitivity CRP, IL-6 = interleukin 6, MMP-8 = matrix metalloproteinase 8, GlycA = glycoprotein acetylation, IGFBP-1 = insulin-like growth factor-binding protein 1. *p<0.05, **p<0.01, #p<0.0045 (Bonferroni).

n-values for GlycA at baseline were total gestational weight gain: 207, late gestational weight gain: 208, preeclampsia or gestational hypertension: 208 (n with event: 18), length of gestation: 208, induction of labor: 208 (n with event: 94), cesarean section: 208 (n with event: 30), birth weight: 204, birth weight <10^th^ percentile: 204 (n with event: 19), birth weight >90^th^ percentile: 204 (n with event: 32), NICU admission: 207 (n with event: 70), newborn i.v. glucose: 206 (n with event: 48).

**Supplementary table 2b – Associations of inflammatory markers and IGFBP-1 concentrations at 36 gestational weeks with maternal and neonatal outcomes**

|  | | **Maternal outcomes** | | | | | |  | **Neonatal outcomes** | | | | | |
| --- | --- | --- | --- | --- | --- | --- | --- | --- | --- | --- | --- | --- | --- | --- |
|  | | **Total gestational weight gain** | **Late gestational weight gain** | **Preeclampsia or gestational hypertension** | **Length of gestation** | **Induction of labor** | **Cesarean section** |  | **Birth weight** | **Birth weight <10^th^ percentile** | **Birth weight >90^th^ percentile** | | **NICU admission** | **Newborn I.V. glucose** |
|  |  | *kg/SD* | *kg/SD* | *OR/SD* | *weeks/SD* | *OR/SD* | *OR/SD* |  | *SD/SD* | *OR/SD* | *OR/SD* | | *OR/SD* | *OR/SD* |
|  | total n:  n with event: | *188* | *189* | *189*  *19* | *189* | *189*  *91* | *189*  *26* |  | *185* | *185*  *19* | *185*  *25* | | *188*  *58* | *187*  *39* |
| **Inflammation at 36 gw** | |  |  |  |  |  |  |  |  |  |  | |  |  |
|  | hsCRP | -0.13 [-0.8; 0.76] | -0.065 [-0.42; 0.41] | 0.62 [0.22; 1.1] | 0.0032 [-0.23; 0.21] | 1.2 [0.85; 1.6] | 0.85 [0.47; 1.4] |  | -0.042 [-0.17; 0.13] | 1.1 [0.72; 1.9] | 0.96 [0.63; 1.4] | | 1.2 [0.78; 1.6] | 1.4 [0.87; 2] |
|  | IL-6 | 0.51 [-0.061; 1.8] | 0.11 [-0.49; 0.48] | 1.5 [0.7; 2.4] | -0.19 [-0.44; -0.03] | 1.1 [0.72; 1.6] | 0.96 [0.57; 1.7] |  | -0.04 [-0.16; 0.2] | 1.6 [0.81; 3] | | 0.99 [0.58; 1.8] | 1.1 [0.81; 2] | 1.2 [0.87; 2.4] |
|  | MMP-8 | -0.63 [-1.2; 0.11] | 0.11 [-0.27; 0.66] | 1 [0.48; 1.8] | 0.011 [-0.18; 0.22] | 1.2 [0.86; 1.7] | 1.3 [0.9; 2.3] |  | -0.17 [-0.34; -0.037]* | 1.3 [0.94; 2.3] | | 0.65 [0.33; 1] | 0.84 [0.56; 1.2] | 0.93 [0.57; 1.4] |
|  | GlycA | -0.15 [-0.86; 0.59] | -0.22 [-0.57; 0.15] | 1.2 [0.81; 1.9] | -0.12 [-0.31; 0.11] | 1.3 [0.95; 1.7] | 1.1 [0.69; 1.6] |  | -0.033 [-0.17; 0.1] | 1.2 [0.68; 2] | | 0.66 [0.43; 0.96] | 0.98 [0.69; 1.4] | 1.1 [0.76; 1.5] |
| **IGFBP-1 at 36 gw** | |  |  |  |  |  |  |  |  |  | |  |  |  |
|  | Non-phosphorylated | -1.1 [-1.8; -0.52]**# | -0.55 [-0.96; -0.21]** | 0.93 [0.47; 1.6] | 0.013 [-0.26; 0.23] | 0.84 [0.52; 1.2] | 0.49 [0.24; 0.84]* |  | -0.14 [-0.31; -0.025] | 1.1 [0.73; 2.1] | | 0.58 [0.28; 1] | 1.2 [0.74; 1.6] | 1.1 [0.6; 1.6] |
|  | Low-phosphorylated | -0.66 [-1.4; 0.37] | -0.37 [-0.71; 0.036] | 0.97 [0.5; 1.6] | -0.14 [-0.34; 0.096] | 0.82 [0.61; 1.1] | 0.68 [0.36; 1.1] |  | -0.044 [-0.19; 0.11] | 1.1 [0.64; 1.9] | | 0.78 [0.44; 1.3] | 0.9 [0.61; 1.3] | 0.92 [0.55; 1.4] |
|  | High-phosphorylated | 0.23 [-0.52; 1.1] | -0.23 [-0.61; 0.16] | 0.97 [0.53; 1.7] | 0.073 [-0.13; 0.29] | 0.89 [0.65; 1.2] | 0.78 [0.48; 1.2] |  | -0.018 [-0.16; 0.13] | 1.4 [0.8; 2.4] | | 1 [0.64; 1.6] | 0.88 [0.61; 1.2] | 0.95 [0.61; 1.4] |

Both metformin and insulin treated patients were included. Measures are expressed as odds ratios (OR) or regression β-estimates with 95% confidence intervals. Birth weight was measured in population SD units. SD = standard deviation, NICU = neonatal intensive care unit, i.v. = intravenous, hsCRP = high sensitivity CRP, IL-6 = interleukin 6, MMP-8 = matrix metalloproteinase 8, GlycA = glycoprotein acetylation, IGFBP-1 = insulin-like growth factor-binding protein 1. *p<0.05, **p<0.01, #p<0.0045 (Bonferroni).

n-values for GlycA at 36 gw total gestational weight gain: 197, late gestational weight gain: 198, preeclampsia or gestational hypertension: 198 (n with event: 20), length of gestation: 198, induction of labor: 198 (n with event: 94), cesarean section: 198 (n with event: 30), birth weight: 194, birth weight <10^th^ percentile: 194 (n with event: 20), birth weight >90^th^ percentile: 194 (n with event: 29), NICU admission: 197 (n with event: 62), newborn i.v. glucose: 196 (n with event: 42).
