# Supplementary material for: Metformin and insulin treatment of gestational diabetes: effects on inflammatory markers and IGF-binding protein-1 – secondary analysis of a randomized controlled trial
Source: BMC Pregnancy Childbirth. 2020 Jul 11;20:401. doi: 10.1186/s12884-020-03077-6 (PMC7353798; doi:10.1186/s12884-020-03077-6)
Supplement: Supplementary file 4 — Additional file 4: Table S3. Associations of inflammatory markers and IGFBP-1 concentrations at baseline and 36 gestational weeks with clinical outcomes adjusted for pre-pregnancy BMI in metformin and insulin treated patients combined. [file 12884_2020_3077_MOESM4_ESM.docx]

**Supplementary table 3a – Associations of inflammatory markers and IGFBP-1 concentrations at baseline with maternal and neonatal outcomes adjusted for pre-pregnancy BMI in metformin and insulin treated patients combined**

|  | | **Maternal Outcomes** | | | | | |  | **Neonatal Outcomes** | | | | |
| --- | --- | --- | --- | --- | --- | --- | --- | --- | --- | --- | --- | --- | --- |
|  | | **Total gestational weight gain** | **Late gestational weight gain** | **Preeclampsia or gestational hypertension** | **Length of gestation** | **Induction of labor** | **Cesarean section** |  | **Birth weight** | **Birth weight <10^th^ percentile** | **Birth weight >90^th^ percentile** | **NICU admission** | **Newborn I.V. glucose** |
|  |  | *kg/SD* | *kg/SD* | *OR/SD* | *weeks/SD* | *OR/SD* | *OR/SD* |  | *SD/SD* | *OR/SD* | *OR/SD* | *OR/SD* | *OR/SD* |
|  | total n:  n with event: | *201* | *202* | *202*  *17* | *202* | *202*  *92* | *202*  *29* |  | *198* | *198*  *17* | *198*  *30* | *201*  *67* | *200*  *45* |
| **Inflammation at baseline** | |  |  |  |  |  |  |  |  |  |  |  |  |
|  | hsCRP | 0.72 [0.55; 1.5]* | 0.21 [-0.22; 0.64] | 0.91 [0.27; 1.3] | 0.2 [0.035; 0.37]* | 0.73 [0.45; 0.85] | 0.98 [0.6; 1.4] |  | -0.089 [-0.27; 0.0033] | 1.2 [0.7; 2.1] | 0.74 [0.35; 0.93] | 0.94 [0.65; 1.3] | 1 [0.6; 1.4] |
|  | IL-6 | 0.15 [-0.31; 1.1] | -0.065 [-0.48; 0.39] | 1.0 [0.6; 1.5] | 0.0097 [-0.18; 0.17] | 0.77 [0.52; 0.96] | 1.1 [0.68; 1.6] |  | 0.01 [-0.13; 0.13] | 0.87 [0.43; 1.4] | 0.99 [0.62; 1.3] | 1 [0.73; 1.4] | 1 [0.71; 1.4] |
|  | MMP-8 | 0.31 [-0.074; 1.3] | 0.43 [0.054; 0.8]* | 1.3 [0.43; 2] | 0.014 [-0.18; 0.19] | 0.95 [0.58; 1.2] | 1.3 [0.89; 1.8] |  | -0.041 [-0.2; 0.053] | 0.99 [0.59; 1.6] | 0.81 [0.4; 1.2] | 1.2 [0.84; 1.6] | 1.1 [0.75; 1.5] |
|  | GlycA | 0.037 [-0.52; 0.92] | -0.32 [-0.75; 0.06] | 1.5 [0.93; 2.2] | -0.18 [-0.37; -0.0049] | 1.1 [0.81; 1.4] | 1.2 [0.84; 1.6] |  | 0.029 [-0.12; 0.16] | 1.1 [0.69; 2] | 0.9 [0.6; 1.2] | 1.1 [0.79; 1.4] | 1.1 [0.79; 1.5] |
| **IGFBP-1 at baseline** | |  |  |  |  |  |  |  |  |  |  |  |  |
|  | Non-phosphorylated | -1.5 [-3; -1.2]**# | -0.47 [-0.91; -0.17]* | 0.97 [0.48; 2.4] | 0.076 [-0.18; 0.28] | 0.94 [0.64; 1.3] | 0.57 [0.3; 1] |  | -0.14 [-0.26; -0.0071]* | 1.1 [0.66; 2.2] | 0.64 [0.39; 1.2] | 1.2 [0.8; 1.6] | 1.1 [0.69; 1.8] |
|  | Low-phosphorylated | -1 [-2.1; -0.64]**# | -0.37 [-0.69; -0.052] | 1.0 [0.52; 2] | -0.035 [-0.26; 0.17] | 0.89 [0.69; 1.3] | 0.83 [0.48; 1.4] |  | -0.051 [-0.15; 0.082] | 1 [0.59; 1.6] | 0.72 [0.48; 1.2] | 0.89 [0.63; 1.2] | 0.91 [0.59; 1.4] |
|  | High-phosphorylated | -0.46 [-1.3; -0.38] | -0.38 [-0.74; -0.083] | 0.88 [0.63; 1.7] | -0.049 [-0.23; 0.15] | 0.75 [0.61; 1.2] | 0.76 [0.53; 1.2] |  | 0.02 [-0.058; 0.2] | 0.7 [0.37; 1.1] | 0.86 [0.66; 1.4] | 1 [0.76; 1.4] | 1.1 [0.81; 1.7] |

Both metformin and insulin treated patients were included. Measures are expressed as odds ratios (OR) or regression β-estimates with 95% confidence intervals. Birth weight was measured in population SD units. SD = standard deviation, NICU = neonatal intensive care unit, i.v. = intravenous, hsCRP = high sensitivity CRP, IL-6 = interleukin 6, MMP-8 = matrix metalloproteinase 8, GlycA = glycoprotein acetylation, IGFBP-1 = insulin-like growth factor-binding protein 1. *p<0.05, **p<0.01, #p<0.0045 (Bonferroni).

n-values for GlycA at baseline were total gestational weight gain: 207, late gestational weight gain: 208, preeclampsia or gestational hypertension: 208 (n with event: 18), length of gestation: 208, induction of labor: 208 (n with event: 94), cesarean section: 208 (n with event: 30), birth weight: 204, birth weight <10^th^ percentile: 204 (n with event: 19), birth weight >90^th^ percentile: 204 (n with event: 32), NICU admission: 207 (n with event: 70), newborn i.v. glucose: 206 (n with event: 48).

**Supplementary table 3b – Associations of inflammatory markers and IGFBP-1 concentrations at 36 gestational weeks with maternal and neonatal outcomes adjusted for pre-pregnancy BMI in metformin and insulin treated patients combined**

|  | | **Maternal Outcomes** | | | | | |  | **Neonatal Outcomes** | | | | |
| --- | --- | --- | --- | --- | --- | --- | --- | --- | --- | --- | --- | --- | --- |
|  | | **Total gestational weight gain** | **Late gestational weight gain** | **Preeclampsia or gestational hypertension** | **Length of gestation** | **Induction of labor** | **Cesarean section** |  | **Birth weight** | **Birth weight <10^th^ percentile** | **Birth weight >90^th^ percentile** | **NICU admission** | **Newborn I.V. glucose** |
|  |  | *kg/SD* | *kg/SD* | *OR/SD* | *weeks/SD* | *OR/SD* | *OR/SD* |  | *SD/SD* | *OR/SD* | *OR/SD* | *OR/SD* | *OR/SD* |
|  | total n:  n with event: | *188* | *189* | *189*  *19* | *189* | *189*  *91* | *189*  *26* |  | *185* | *185*  *19* | *185*  *25* | *188*  *58* | *187*  *39* |
| **Inflammation at 36 gw** | |  |  |  |  |  |  |  |  |  |  |  |  |
|  | hsCRP | 0.084 [-0.5; 1.3] | -0.077 [-0.44; 0.37] | 0.52 [0.088; 0.9] | 0.0012 [-0.23; 0.21] | 1.1 [0.71; 1.4] | 0.79 [0.36; 1.2] |  | -0.054 [-0.2; 0.091] | 1.1 [0.7; 1.9] | 0.9 [0.53; 1.2] | 1.2 [0.79; 1.6] | 1.3 [0.83; 1.9] |
|  | IL-6 | 0.42 [-0.46; 1.3] | 0.12 [-0.47; 0.5] | 1.5 [0.89; 3] | -0.19 [-0.44; -0.03] | 1.2 [0.83; 1.8] | 0.98 [0.6; 1.8] |  | -0.036 [-0.15; 0.22] | 1.6 [0.83; 3.1] | 1 [0.61; 1.9] | 1.1 [0.81; 2] | 1.2 [0.88; 2.5] |
|  | MMP-8 | -0.44 [-0.87; 0.61] | 0.1 [-0.28; 0.64] | 0.97 [0.37; 1.6] | 0.0098 [-0.19; 0.22] | 1.2 [0.74; 1.5] | 1.3 [0.84; 2.2] |  | -0.18 [-0.38; -0.063]* | 1.3 [0.93; 2.3] | 0.6 [0.25; 0.93] | 0.84 [0.56; 1.2] | 0.91 [0.54; 1.4] |
|  | GlycA | -0.29 [-1.2; 0.28] | -0.21 [-0.56; 0.16] | 1.3 [0.96; 2.3] | -0.11 [-0.31; 0.11] | 1.3 [1; 1.9] | 1.1 [0.75; 1.8] |  | -0.027 [-0.16; 0.11] | 1.2 [0.71; 2] | 0.68 [0.46; 1] | 0.98 [0.69; 1.4] | 1.1 [0.78; 1.6] |
| **IGFBP-1 at 36 gw** | |  |  |  |  |  |  |  |  |  |  |  |  |
|  | Non-phosphorylated | -1.5 [-2.9; -1.3]**# | -0.56 [-0.97; -0.22]** | 1 [0.63; 2.3] | 0.016 [-0.25; 0.24] | 0.92 [0.67; 1.4] | 0.54 [0.31; 0.97] |  | -0.13 [-0.26; 0.0094] | 1.1 [0.74; 2.2] | 0.64 [0.37; 1.2] | 1.2 [0.75; 1.6] | 1.1 [0.66; 1.7] |
|  | Low-phosphorylated | -0.99 [-2.1; -0.45]** | -0.37 [-0.7; 0.044] | 1.1 [0.66; 2] | -0.14 [-0.34; 0.091] | 0.89 [0.72; 1.4] | 0.72 [0.45; 1.2] |  | -0.028 [-0.16; 0.14] | 1.1 [0.65; 1.9] | 0.83 [0.52; 1.5] | 0.9 [0.6; 1.3] | 0.94 [0.59; 1.4] |
|  | High-phosphorylated | -0.3 [-1.6; -0.037] | -0.23 [-0.6; 0.16] | 1.1 [0.77; 2.4] | 0.086 [-0.11; 0.32] | 1 [0.88; 1.6] | 0.84 [0.58; 1.4] |  | 0.0093 [-0.11; 0.19] | 1.5 [0.9; 2.6] | 1.2 [0.83; 2.1] | 0.87 [0.59; 1.2] | 0.99 [0.67; 1.5] |

Both metformin and insulin treated patients were included. Measures are expressed as odds ratios (OR) or regression β-estimates with 95% confidence intervals. Birth weight was measured in population SD units. SD = standard deviation, NICU = neonatal intensive care unit, i.v. = intravenous, hsCRP = high sensitivity CRP, IL-6 = interleukin 6, MMP-8 = matrix metalloproteinase 8, GlycA = glycoprotein acetylation, IGFBP-1 = insulin-like growth factor-binding protein 1. *p<0.05, **p<0.01, #p<0.0045 (Bonferroni).

n-values for GlycA at 36 gw total gestational weight gain: 197, late gestational weight gain: 198, preeclampsia or gestational hypertension: 198 (n with event: 20), length of gestation: 198, induction of labor: 198 (n with event: 94), cesarean section: 198 (n with event: 30), birth weight: 194, birth weight <10^th^ percentile: 194 (n with event: 20), birth weight >90^th^ percentile: 194 (n with event: 29), NICU admission: 197 (n with event: 62), newborn i.v. glucose: 196 (n with complication: 42).
