# Supplementary material for: Metformin and insulin treatment of gestational diabetes: effects on inflammatory markers and IGF-binding protein-1 – secondary analysis of a randomized controlled trial
Source: BMC Pregnancy Childbirth. 2020 Jul 11;20:401. doi: 10.1186/s12884-020-03077-6 (PMC7353798; doi:10.1186/s12884-020-03077-6)
Supplement: Supplementary file 5 — Additional file 5: Table S4. Regression models with significant (p < 0.05) interaction between treatment group (metformin or insulin) and the association between outcome and the independent variable. [file 12884_2020_3077_MOESM5_ESM.docx]

**Supplementary table 4 –** **Regression models with significant (p<0.05) interaction between treatment group (metformin or insulin) and the association between outcome and the independent variable.**

| **Independent variable** | | **Outcome** | **Model** | **p-value for interaction** | **Insulin** | **Metformin** |
| --- | --- | --- | --- | --- | --- | --- |
| **Baseline** | |  |  |  |  |  |
|  | low-pIGFBP-1 | preeclampsia or gestational hypertension OR/SD | model 0 | 0.044 | 1.2 [0.58; 2.1] (0.56) | 0.11 [0.01; 0.77] (0.053) |
|  | high-pIGFBP-1 | preeclampsia or gestational hypertension OR/SD | model 0 | 0.039 | 1.2 [0.67; 2.2] (0.53) | 0.28 [0.06; 0.56] (0.045) |
|  | low-pIGFBP-1 | induction of labor OR/SD | model 0 | 0.018 | 0.97 [0.65; 1.4] (0.86) | 0.38 [0.17; 0.74] (0.0066) |
|  | hsCRP | NICU admission OR/SD | model 0 | 0.021 | 0.59 [0.34; 1] (0.053) | 1.3 [0.8; 1.9] (0.20) |
|  | hsCRP | NICU admission OR/SD | model 1 | 0.021 | 0.59 [0.33; 0.99] (0.052) | 1.3 [0.76; 1.9] (0.24) |
| **36 gestational weeks** | |  |  |  |  |  |
|  | MMP-8 | late GWG (kg/SD) | model 0 | 0.024 | -0.24 [-0.82; 0.56] (0.35) | 0.74 [0.18; 1.4] (0.035) |
|  | MMP-8 | late GWG (kg/SD) | model 1 | 0.026 | -0.24 [-0.84; 0.54] (0.35) | 0.73 [0.16; 1.3] (0.039) |
|  | high-pIGFBP-1 | preeclampsia or gestational hypertension OR/SD | model 0 | 0.037 | 1.5 [0.65; 3.3] (0.21) | 0.41 [0.13; 1.2] (0.092) |
|  | hsCRP | length of gestation (weeks/SD) | model 0 | 0.017 | -0.16 [-0.59; 0.024] (0.18) | 0.4 [0.049; 0.68] (0.046) |
|  | hsCRP | length of gestation (weeks/SD) | model 1 | 0.018 | -0.16 [-0.58; 0.027] (0.19) | 0.41 [0.06; 0.69] (0.048) |
|  | non-pIGFBP-1 | induction of labor OR/SD | model 1 | 0.037 | 1.1 [0.58; 1.7] (0.63) | 0.49 [0.22; 0.88] (0.030) |

Data is given as regression β-estimates or odds ratios (OR) [95% confidence interval] (p-value). Model 0: unadjusted, model 1: adjusted for pre-pregnancy BMI. NICU = neonatal intensive care unit, GWG = (maternal) gestational weight gain, pIGFBP-1 = phosphorylated insulin-like growth factor-binding protein 1, MMP-8 = matrix metalloproteinase 8, hsCRP = high sensitivity CRP. None of the p-values were below Bonferroni adjusted threshold of 0.0045.
